# Psychological inflexibility and valuing happiness: Dangerous liaisons

**DOI:** 10.3389/fpsyg.2022.949615

**Published:** 2022-08-11

**Authors:** Sonsoles Valdivia-Salas, A. Sebastian Lombas, Sonia Salvador, Ginesa López-Crespo

**Affiliations:** ^1^Department of Psychology and Sociology, University of Zaragoza, Teruel, Spain; ^2^Aragon Institute for Health Research (Instituto de Investigación Sanitaria de Aragón), Zaragoza, Spain

**Keywords:** valuing happiness, psychological inflexibility, cognitive fusion, experiential avoidance, positive affect, negative affect, adults

## Abstract

Previous evidence has shown that excessive valuing happiness may relate to lower psychological wellbeing across cultures. Considering the lack of data with Spanish population, we examined the relation between tightly holding happiness emotion goals and subjective wellbeing in a sample of Spanish women, and explored the mediation role exerted by psychological inflexibility components (namely, cognitive fusion and experiential avoidance) in the relation between valuing happiness and subjective wellbeing. A female adult sample (*n* = 168) filled out measures of excessive valuing happiness, psychological inflexibility, positive affect, negative affect, and life satisfaction. Valuing happiness only showed positive total effects on negative affect and strong direct effects on both cognitive fusion and experiential avoidance. Analyses revealed the mediating roles exerted by psychological inflexibility components, with experiential avoidance leading to lower pleasure; and cognitive fusion leading to greater displeasure and lower life satisfaction. Psychological inflexibility components explained between 40 and 80% of the total effect of valuing happiness on our outcome variables. Our findings highlight the need for further research on the benefits of hedonic vs. values-based approaches to happiness.

## Introduction

Traditionally, happiness has been found in two different aspects of life in what is known as hedonic and eudaimonic approaches to happiness (Waterman, 1993). In the hedonic tradition, happiness is defined as the experience of high levels of pleasant emotions and moods, low levels of negative emotions and moods, and life satisfaction (Diener, 1984). In the eudamonic tradition, however, happiness occurs when people's life activities are congruent with their true self and held values, and are fully engaged (Waterman, 1993). Within the hedonic tradition, research has shown that positive emotions have a positive impact on our lives, facilitating thought–action repertoires; building vital social, physical, and cognitive resources; leading to more prosocial outcomes and enhanced affiliation; and improving physical and mental health (e.g., Bastian et al., 2014; Catalino et al., 2014; Luhmann et al., 2016). Research also shows, however, that obsessively pursuing hedonic happiness or tightly holding happiness emotion goals, what has been termed valuing happiness (Mauss et al., 2011), might relate to lower hedonic balance and psychological wellbeing, poor life satisfaction, and more feelings of loneliness (Mauss et al., 2011, 2012), depression (Catalino et al., 2014), and borderline symptomatology (Ford et al., 2015), also in adolescents (Gentzler et al., 2019). Independent researchers employing the Values Happiness Scale (Mauss et al., 2011) have reported that the detrimental outcomes of holding happiness emotion goals occur under specific conditions: within individualistic cultures where happiness is pursued in socially-disenganged independent ways (Ford et al., 2015; Wong et al., 2020); in individuals who experience negative emotions in cultural contexts where positive emotions are highly valued (Bastian et al., 2014) and who show poor mood regulation skills (Fergus and Bardeen, 2016; Clauss et al., 2019; Kahriz et al., 2019); and also, when individuals score high in neuroticism and regard happiness as an essentially fragile mood state that can easily turn into less favorable states and feelings (Joshanloo, 2018). Placing extreme importance to experiencing happiness in our lives, or holding irrational happiness beliefs, i.e., those that include words such as “must”, “should”, “ought to” (Yildirim, 2021; Yildirim and Maltby, 2022), may lead to constant self-monitoring of our mood state and the deliberate avoidance or denial of current unpleasant emotions that are deemed incompatible with the desired happy mood (Gruber et al., 2011). It is reasonable to believe, hence, that avoidance could be contributing to the detrimental consequences of valuing happiness. Indeed, worrying about the possibility of being unhappy seems key in the relation between valuing happiness and lower wellbeing (Luhmann et al., 2016; Joshanloo, 2018).

Avoidance is central to the psychological inflexibility model of psychopathology (Hayes et al., 1996, 2004). Psychological inflexibility refers to the tendency to down-regulate unwanted thoughts and feelings (Hayes et al., 1996; Bond et al., 2011), and becomes life constricting when it occurs across life domains and regardless of contextual circumstances that suggest its utilization is unworkable. Psychological inflexibility is a consistent predictor of daily anxiety-related symptoms and emotional distress, diminished positive life appraisals and emotions, and meaning in life (e.g., Kashdan and Breen, 2007; Machell et al., 2015), to the point that it is regarded as a transdiagnostic process (Monestès et al., 2018).

Psychological inflexibility reflects six interrelated processes (Hayes et al., 2004), chief among them, cognitive fusion and experiential avoidance (McCracken et al., 2004; Greco et al., 2008; Valdivia-Salas et al., 2017). Cognitive fusion is the process by which thoughts are taken literally so that, instead of experiencing them as mental events that do not necessarily need to be acted upon, the person is dominated by or entangled with them (Gillanders et al., 2014). Experiential avoidance is the tendency to fight against unwanted thoughts and feelings to change their content or frequency (Hayes et al., 1996).

In the present study, we tested two predictions. First, bearing in mind that valuing happiness may lead to the avoidance of unpleasant experiences, we expected a positive relation between valuing happiness and psychological inflexibility components. As well, based on previous studies that have linked psychological inflexibility to more emotional distress and less positive life appraisals (e.g., Kashdan and Breen, 2007; Machell et al., 2015), we expected a negative relation between psychological inflexibility components and wellbeing. Consequently, we tested the mediation role of psychological inflexibility components in the negative relation between valuing happiness and psychological wellbeing.

## Methods

### Participants

A total of 203 volunteers (88.7% women; 11.3% men; *M* age = 36, *SD* = 14.68) participated in the study. The inclusion criteria were: (1) being 18 years old or older, and (2) being fluent in Spanish. Considering the exploratory nature of the study, we employed a convenience sampling method. Researchers involved in the study distributed an online questionnaire among their personal and social network contacts, and all interested participants meeting the inclusion criteria filled out the questionnaires online.

Exploratory data analyses yielded different effect sizes as a matter of gender. Given the small number of male participants, which precluded performing analysis with enough power, and the existence of previous similar studies with female-only samples (Shallcross et al., 2010; Mauss et al., 2011), we reconducted the analyses with women. Thus, our final sample consisted of 180 women with ages ranging between 18 and 76 years old (*M* = 35.5, *SD* = 14.50).

### Variables and instruments

#### Valuing happiness

We administered an *ad hoc* Spanish translation of the Valuing Happiness Scale (Mauss et al., 2011), for which we employed the parallel back-translation procedure (Brislin, 1986). The items were first translated from English into Spanish by expert translators. The items were then back-translated into English and compared with the original ones. Finally, three experts and five students evaluated the adequacy of the items to the construct being assessed. We found no major difficulties with the semantic equivalence of the items in Spanish. The instrument includes seven items (e.g., “how happy I am at any given moment says a lot about how worthwhile my life is” “I worry about my happiness even when I am happy”), and participants rate how much they agree with each sentence by using a Likert scale ranging from 1 (*strongly disagree*) to 7 (*strongly agree*). The original validation of the scale indicated an adequate internal consistency (alpha = 0.76) and no gender differences, *t*_(290)_ = 0.77, *p* = 0.38. A confirmatory factor analysis in our sample yielded a one-factor solution with good fit to the data in most fit indexes [χ^2^_(df = 11)_ = 31.32, *p* < 0.01; RMSEA = 0.10; CFI = 0.92; SRMR = 0.05], after correlating item 4 error with those of items 1, 2, and 6. Cronbach's alpha in our sample was 0.73.

#### Psychological inflexibility

We administered the Spanish validation of the Avoidance and Fusion Questionnaire for Youth (AFQ-Y; Valdivia-Salas et al., 2017). It includes 17 items assessing cognitive fusion (e.g., “I am afraid of my feelings”); and experiential avoidance (e.g., “I must get rid of my worries and fears so I can have a good life”). Participants rate how true each statement is for them by using a Likert scale ranging from 0 (*not at all true*) to 4 (*very true*). In the original validation study, alpha values for the cognitive fusion and experiential avoidance items were 0.81 and 0.76, respectively. Cronbach's alpha in our sample was 0.81 for both components. We employed the youth version because the wording of the items seems to facilitate its understanding better than the adults version and there is evidence that it works with adults as good as the adults version (Fergus et al., 2012; Corman et al., 2019).

#### Positive and negative affect

We administered the Spanish validation of the Positive and Negative Affect Scale (PANAS; Sandín et al., 1999). Each scale includes two sets of 10 adjectives each, which assess positive affect (e.g., interested and calm) and negative affect (e.g., upset and distressed), respectively. Participants rate how they have felt during the last week by using a Likert scale ranging from 1 (*very slightly or not at all*) to 5 (*extremely*). In the Spanish validation, alpha values for positive affect and negative affect were 0.87 and 0.89, respectively, in women. Cronbach's alpha in our sample was 0.84 for positive affect and 0.88 for negative affect.

#### Life satisfaction

We administered the Spanish validation of the Satisfaction With Life Scale (Atienza et al., 2000). This scale includes five items (e.g., “In most ways my life is close to my ideal”) and participants rate how much they agree with each sentence by using a Likert scale ranging from 1 (*strongly disagree*) to 7 (*strongly agree*). Cronbach's alpha in the original study was 0.84 and in our sample was 0.80.

### Procedure

After selecting the questionnaires to administer, they were uploaded to an online platform and distributed among the researchers personal contacts and social network. Interested participants were first asked for their gender, age and fluency in Spanish. Responders meeting the inclusion criteria proceeded to read and give their informed consent and on to the questionnaires. Responders not meeting the inclusion criteria were dismissed and fully debriefed. All participants met the inclusion criteria.

## Data analyses and results

We employed IBM SPSS Statistics software, version 24, to conduct all statistical analyses. Statistical significance was set at *p* < 0.05. In order to examine the mediating role of cognitive fusion and experiential avoidance in the relation between valuing happiness and indexes of subjective wellbeing such as positive affect, negative affect and life satisfaction, we run several mediation analyses with the INDIRECT macro (Preacher and Hayes, 2008). With the purpose of testing the statistical reliability of the indirect effects, we performed the Sobel (1982) test with 95% confidence interval of the mediated effect, calculated with the bias-corrected and accelerated bootstrap method (*n* = 5,000 resamples). According to Fritz and MacKinnon (2007), when the values of standardized coefficients involved in a mediation analysis conducted by using bias-corrected bootstrap method are 0.26 or higher, statistical power of 0.8 can be reached with a sample size of 148. Given that our sample is 180, we expect to have enough statistical power to detect indirect effect sizes with values small and medium. All multiple linear regression analyses conducted prior to calculating the mediated effects met the required assumptions (see MacKinnon, 2008). Effect size magnitudes were interpreted according to Cohen (1988) guidelines. As an additional effect size index, we calculated the proportion of the total effect which was explained by the mediated effect, using absolute values (Alwin and Hauser, 1975).

**Figure 1:**
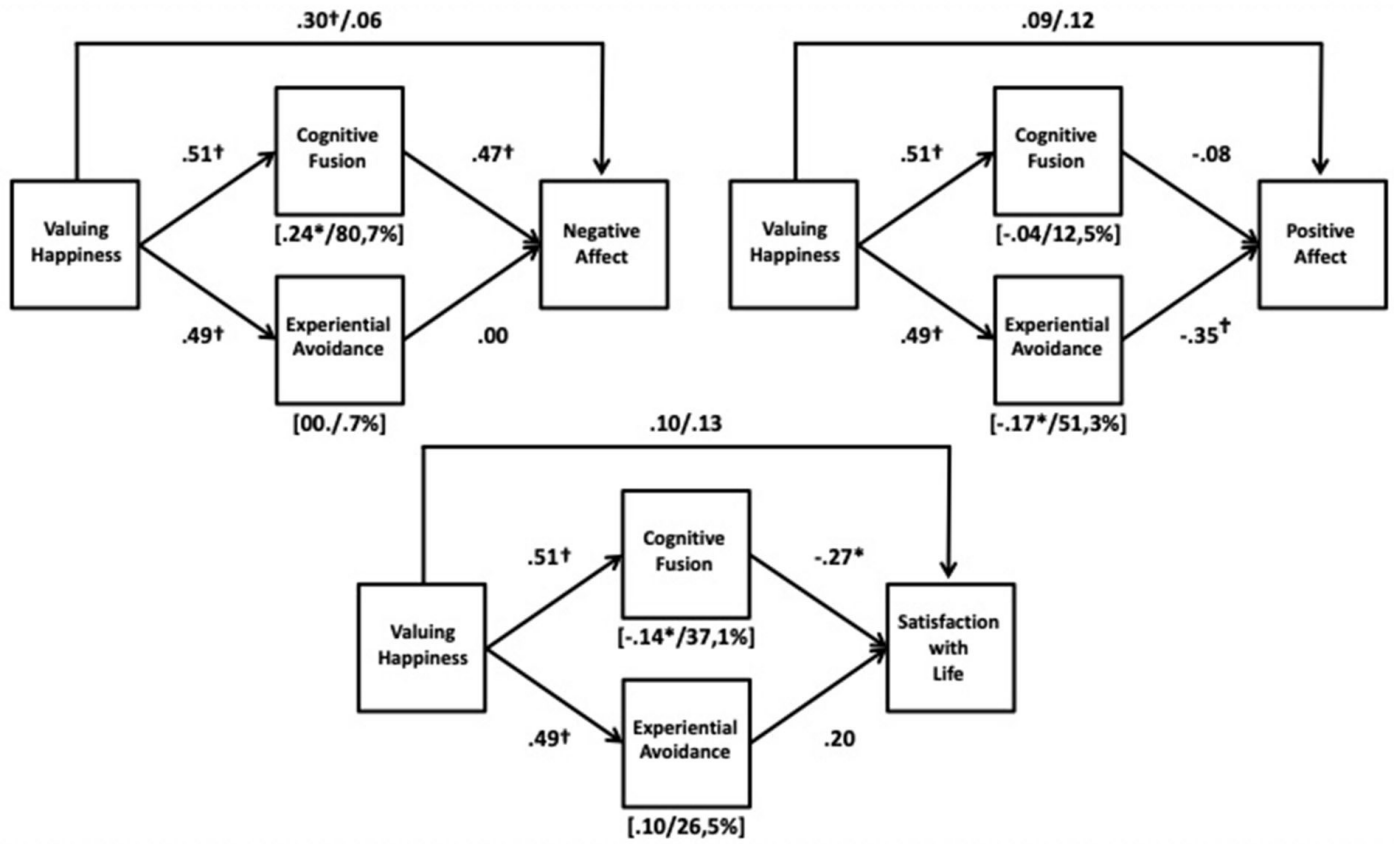
Path diagrams for the mediation between valuing happiness and wellbeing variables through psychological inflexibility components. Regression coefficients were standardized values. Values that are separated by a slash above an arrow indicate total effect (left value) and direct effect (right value). Values in square brackets below a mediator and separated by a slash indicate its indirect effect (left value) and the proportion between its mediated effect and the total effect (right value). *p < 0.05; ^†^*p* < 0.01.

Descriptives and correlations among the variables are shown in Table 1. As shown in Table 2 and in the Figure 1, the total effect of valuing happiness was positive and significant on negative affect, with medium effect size (β = 0.30). In contrast, the total effect on positive affect and life satisfaction did not reach statistical reliability. Similarly, valuing happiness did not produce significant direct effects on any outcome variable.

**Table 1:** Bivariate correlations, means, and standard deviations.

**Variable**	**VH**	**CF**	**EA**	**NA**	**PA**	**SL**
VH						
CF	0.51**					
EA	0.49**	0.80**				
NA	0.30**	0.50**	0.41**			
PA	−0.09	−0.30**	−0.35*	−0.28**		
SL	0.10	−0.04	0.05	0.18*	0.15*	
Mean	4.1	1.6	1.9	2.7	3.3	3.9
*SD*	1.07	0.83	0.81	0.83	0.63	0.67

VH, Valuing Happiness; CF, Cognitive Fusion; EA, Experiential Avoidance; NA, Negative Affect; PA, Positive Affect; SL, Satisfaction with Life; SD, Standard Deviation. *p < 0.05; **p < 0.01.

**Table 2:** Direct and indirect effects of mediational models.

**Mediation model**	**Path**	**Effect**	**95% C.I**.	
			**LL**	**UL**
VH → PI → NA	VH → NA	0.06	–	–
	VH → CF	0.51*	–	–
	VH → EA	0.49*	–	–
	CF → NA	0.47*	–	–
	EA → NA	0.00	–	–
	VH → CF → NA	0.24*	0.12	0.38
	VH → EA → NA	0.00	−0.11	0.13
VH → PI → PA	VH → PA	0.12	–	–
	VH → CF	0.51*	–	–
	VH → EA	0.49*	–	–
	CF → PA	−0.08	–	–
	EA → PA	−0.35*	–	–
	VH → CF → PA	−0.04	−0.17	0.08
	VH → EA → PA	−0.17*	−0.31	−0.06
VH → PI → SL	VH → SL	0.13	–	–
	VH → CF	0.51*	–	–
	VH → EA	0.49*	–	–
	CF → SL	−0.27*	–	–
	EA → SL	0.20	–	–
	VH → CF → SL	−0.14*	−0.29	−0.00
	VH → EA → SL	0.10	−0.03	0.24

95% C.I., Bootstrapping 95% Confidence interval for indirect effect; LL, Lower limit; UL, Upper limit; VH, Valuing Happiness; PI, Psychological Inflexibility; CF, Cognitive Fusion; EA, Experiential Avoidance; NA, Negative Affect; PA, Positive Affect; SL, Satisfaction with Life; *p < 0.05.

Valuing happiness did have large, positive, and significant direct effects on both cognitive fusion (β = 0.51) and experiential avoidance (β = 0.49), and, in turn, these had significant direct relations with the three outcome measures (β = 0.47 and β = −0.27 for cognitive fusion on negative affect and life satisfaction, respectively; and β = −0.35 for experiential avoidance on positive affect), which led to significant indirect effects of valuing happiness on subjective wellbeing. Specifically, on the one hand, experiential avoidance mediated the relation between valuing happiness and positive affect (β = −0.17). On the other hand, cognitive fusion mediated the relation between valuing happiness and both negative affect (β = 0.24), and life satisfaction (β = −0.14). The proportion of the total effect explained by indirect effects through psychological inflexibility components was near 40% and more than 50% for life satisfaction and positive affect, respectively, and close to 81% for negative affect.

## Discussion

The current study investigated the relation between valuing happiness and subjective wellbeing in a sample of Spanish women, and the mediating role of cognitive fusion and experiential avoidance in such relation. Out of the three indices of wellbeing collected, results only revealed a significant positive total effect of valuing happiness on negative affect. This partially replicates previous findings (e.g., Mauss et al., 2011, 2012; Catalino et al., 2014; Ford et al., 2015), which also reported the detrimental effects of valuing happiness on the other two wellbeing indices. Hence, our findings add to the current discussion on whether holding happiness emotion goals leads to wellbeing or can indeed backfire (for instance, see Catalino et al., 2014; Luhmann et al., 2016). Previous evidence using the Valuing Happiness Scale has yielded disparate results, which calls for further investigation of the variables influencing the relation between extremely valuing happiness and subjective wellbeing. There is evidence, for instance, that being overconcerned about feeling positive emotions may backfire in individualistic cultures (Ford et al., 2015) that highly value happiness (Bastian et al., 2014); or when people tend to search for happiness in socially-disengaged independent ways (Wong et al., 2020); or when they deliberately try to feel more positive in a particular moment instead of seeking out activities and contexts from which positive emotions will naturally arise (Catalino et al., 2014).

In the search for the variables that may explain the links between holding happiness emotional goals and wellbeing, our findings suggest that cognitive fusion and experiential avoidance might be critical. On the one hand, valuing happiness showed strong positive associations with both cognitive fusion and experiential avoidance. This goes in line with the evidence showing the link between holding happiness emotion goals and emotion regulatory efforts such as the use of expressive supression (Fergus and Bardeen, 2016) so as to not experience, or avoid, negative emotions. On the other hand, mediation analyses confirmed that valuing happiness was related to lower wellbeing through the mediation of one of the psychological inflexibility components, with non-negligible explained variances ranging from 40 to 80%. In the three cases, cognitive fusion and experiential avoidance related to poorer subjective wellbeing, this is, to lower pleasure, greater displeasure and less life satisfaction. Indirect effects showed that while getting fused to experienced negative emotions (i.e., cognitive fusion) increases negative affect and decreases life satisfaction; making efforts to avoid emotions that are deemed opposite to the desired happiness emotion (i.e., experiential avoidance), blocks the actual experience of positive affect.

The links between psychological inflexibility and both recurrent negative affect and the blocking of positive affect had been documented (Kashdan et al., 2006; Fledderus et al., 2010; Monestès et al., 2018). With the current findings, however, we take a step forward and suggest that holding happiness emotion goals may have detrimental outcomes because it leads to (1) cognitive fusion to emotions that are incompatible with the desired happy mood and to (2) deliberate efforts to replace those with happiness emotion. Further research will elucidate whether there are other intervening variables such as anxious clinging and experience prolonging, both reflecting efforts to sustain desired affective states, also known as experiential approach (Swails et al., 2016; Yildirim et al., 2022). Experiential approach is a different facet of psychological inflexibility and, likewise, implies responding inflexibly to desired private content (self-statements, emotions, expectations) with the aim of experiencing them (i.e., anxious clinging) for as long as possible (i.e., experience prolonging). According to Swails et al. (2016), anxious clinging seems to serve an avoidant function and, although it has been relatively unexplored, there is evidence of its negative relation with various indexes of wellbeing (for more details, see Swails et al., 2016), also in Spain (Reyes-Martín et al., 2021).

Taking a look at the bigger picture, future research will clarify the relative contribution of the various constructs that are being used to explain the cases in which chasing happiness may backfire, such as irrational happiness beliefs, valuing happiness, psychological inflexibility, and experiential approach, just to mention the most closely related to the relations explored in the present study.

As a clinical note, in societies that promote “positive minds” and throw the idea it is possible to “stay positive” through practicing emotional or experiential control techniques, our results suggests it might be beneficial teaching individuals to (1) notice those societal mandates (i.e., cognitive defusion), (2) discriminate whether adjusting their behavior to them brings joy and fulfillment to their lives; and (3) if not, actively search for alternative ways to naturally bring a sense of happiness that goes beyond the inmediate and purposeful experience of positive emotions (i.e., acceptance). On this regard, we point out to the literature on the protective role exerted by values-based pursuing of happiness (e.g., Plumb et al., 2009), as promoted in contextual therapies such as Acceptance and Commitment Therapy (Hayes et al., 1999). Values are intrinsically meaningful principles for living that contribute to wellbeing when they organize and direct current action (Wilson and Dufrene, 2009; Lundgren et al., 2012). There is evidence, for instance, that making decisions or choices about how to organize day-to-day lives bearing in mind the far-reaching implications these may have on our emotions, is beneficial to our wellbeing (Catalino et al., 2014). And this is because values-based searching for happiness may help letting go of forcing happiness in the moment, and instead focusing on increasing the likelihood of experiencing spontaneously generated positive emotions through the day-to-day commitment to actions important and defining of one's true self and held values (Waterman, 1993; Catalino et al., 2014).

The current study is not without limitations, mainly related to its exploratory nature. Besides employing a widely used method to translate the VHS, future studies should consider using a validated version of the instrument in Spain. The convenience sampling method employed yielded a relatively small sample of mostly women within a wide range of ages. Future studies with larger and more representative samples are necessary to confirm the current findings in Spain. As well, research using longitudinal designs is needed to clarify the directions of the relation among the variables. All in all, this is the first time the mediating role of cognitive fusion and experiential avoidance between valuing happiness and subjective wellbeing is evidenced.

## Data availability statement

The raw data supporting the conclusions of this article will be made available by the authors, without undue reservation.

## Ethics statement

The studies involving human participants were reviewed and approved by Comité de Ética de la Investigación de la Comunidad Autónoma de Aragón (Ethics Committe for Research in Aragon, Spain). The patients/participants provided their written informed consent to participate in this study.

## Author contributions

SV and ASL designed the study. SS and GL supervised the data collection. ASL conducted the data analyses. SS drafted the initial version of the manuscript. SV, ASL, and GL drafted the final version of the manuscript. All authors contributed to the article and approved the submitted version.

## Funding

This research was supported by Government of Aragon (Grant Number S62_20R).

## Conflict of interest

The authors declare that the research was conducted in the absence of any commercial or financial relationships that could be construed as a potential conflict of interest.

## Publisher's note

All claims expressed in this article are solely those of the authors and do not necessarily represent those of their affiliated organizations, or those of the publisher, the editors and the reviewers. Any product that may be evaluated in this article, or claim that may be made by its manufacturer, is not guaranteed or endorsed by the publisher.
